# ﻿Three novel species of *Cladosporium* and *Sarocladium* isolated from palm trees

**DOI:** 10.3897/mycokeys.123.165471

**Published:** 2025-09-24

**Authors:** Lin Zhou, Qiu-Yue Zhang, Yu Wan, Yi-Lin Chen, De-Wei Li, Yun-Hong Tan, Hui Sun, Li-Hua Zhu

**Affiliations:** 1 College of Forestry and Grassland, Nanjing Forestry University, Nanjing 210037, China Nanjing Forestry University Nanjing China; 2 Collaborative Innovation Center of Sustainable Forestry in Southern China, Nanjing-210037, China Collaborative Innovation Center of Sustainable Forestry in Southern China Nanjing China; 3 The Connecticut Agricultural Experiment Station Valley Laboratory, Windsor, CT 06095, USA The Connecticut Agricultural Experiment Station Valley Laboratory Windsor United States of America; 4 Chinese Academy of Sciences & Center for Integrative Conservation, Xishuangbanna Tropical Botanical Gar-den, Chinese Academy of Sciences, Mengla, Yunnan 666303, China Chinese Academy of Sciences Mengla China

**Keywords:** Ascomycota, biodiversity, phylogeny, systematics, taxonomy

## Abstract

Palm trees (Arecaceae) are among the most widely used ornamental plants in southern China. In this study, samples of senescent leaves and petioles from four species of palm plants were collected in Yunnan Province, China, and fungal isolations were performed. Species identification was conducted using a phylogenetic approach based on combined sequence data from the internal transcribed spacer (ITS) region, large subunit ribosomal DNA (LSU), actin gene (*ACT*), and translation elongation factor (*TEF*), together with morphological studies. As a result, three new species—*Cladosporium menglunense*, *Sarocladium menglaense*, and *S. yunnanense*—are proposed. By providing detailed morphological and molecular data, this study lays a foundation for future research on species diversity and diseases of palm plants.

## ﻿Introduction

The Arecaceae family comprises approximately 2,800 species across 210 genera, characterized by large leaves that are typically palmately lobed or pinnately compound (Dransfield et al. 2005). Arecaceae species hold significant economic importance, predominantly as ornamental trees in urban landscapes and residential gardens across southern China. However, more than 80% of cultivated palm species in China are introduced taxa, whose aesthetic value is frequently compromised by pathogen-induced diseases (Peng et al. 2015).

The genus *Cladosporium* Link is a species-rich genus in Cladosporiaceae, Cladosporiales, characterized by coronate conidiogenous loci; conidial hila formed by a central convex dome surrounded by a raised periclinal rim (David 1997); and the formation of various conidia, including ramoconidia, secondary ramoconidia, intercalary conidia, and small terminal conidia (Braun et al. 2003; Crous et al. 2007; Schubert et al. 2007; Bensch et al. 2012; Abdollahzadeh et al. 2020). Species of *Cladosporium* are cosmopolitan in distribution and are commonly encountered on all kinds of plants, fungi, and biological debris, and they occupy various ecological niches as saprotrophs (Iturrieta-González et al. 2021), endophytes (Hamayun et al. 2009), phytopathogens (Marin-Felix et al. 2017), animal pathogens (Sandoval-Denis et al. 2016), and hyperparasites (Anderson et al. 2016). Moreover, *Cladosporium* is one of the largest and most heterogeneous genera of hyphomycetes. At present, this genus has ca 350 species (Hyde et al. 2024), which are divided into three species complexes: *Cladosporium cladosporioides*, *C. herbarum*, and *C. sphaerospermum* (Hamayun et al. 2009; Abdollahzadeh et al. 2020; Lee et al. 2023). With increasing attention on *Cladosporium*, new species and new combinations are being proposed continually. Although the internal transcribed spacer (ITS) region has been selected as the primary fungal barcode marker, it provides low resolution for *Cladosporium*. Therefore, for species-level identification of *Cladosporium*, the actin (*ACT*) and translation elongation factor 1 (*TEF*) genes have been proposed (Schubert et al. 2007; Bensch et al. 2012; Lee et al. 2023).

The genus *Sarocladium* belongs to Sarocladiaceae, Hypocreales, and was established by Gams and Hawksworth (1975) with *S. oryzae* (Sawada) W. Gams & D. Hawksw. as the type species and *S. attenuatum* W. Gams & D. Hawksw. as an additional species described at the time of introduction. The genus is characterized by cylindrical conidiogenous phialides arising solitarily from undifferentiated hyphae or conidiophores that are sparsely to densely branched, with ellipsoidal conidia formed in false heads (Summerbell et al. 2011; Ou et al. 2020; Hou et al. 2023). *Sarocladium* presently encompasses 30 species (Hyde et al. 2024), many of which are plant pathogens, opportunistic human pathogens, endophytes, or mycoparasites (Rivera-Varas et al. 2007). The genus is especially well known for its plant pathogenic species, including the causal agent of sheath rot of rice (Ayyadurai et al. 2005).

During research on the fungal diversity of palm species in Yunnan Province, we collected eleven fungal samples that displayed morphological characteristics typical of the genera *Cladosporium* and *Sarocladium*. Our objective was to analyze these samples using multiple genetic markers (ITS + *ACT* + *TEF* for *Cladosporium* and ITS + LSU for *Sarocladium*) to investigate their phylogenetic relationships and to determine their morphological differences in comparison with existing species within their respective genera.

## ﻿Materials and methods

### ﻿Isolation of the potential fungal pathogen

Field surveying and sampling were conducted in November 2024 at the Xishuangbanna Tropical Botanical Garden, Yunnan Province, China. The palm trees (Arecaceae) surveyed comprised four species, with samples collected specifically from senescent fronds (leaves and petioles). The collected specimens are listed in Table 1. The leaves were first rinsed with tap water for 8–12 hours and then placed in a fume hood with absorbent paper for drying. Petri dishes (150 mm) and filter papers of the same size were prepared. After the leaf surface moisture had dried, the leaves were placed into the Petri dishes, sterile water was added to moisten the filter paper, and the dishes were sealed with parafilm, leaving a 1–2 cm opening for ventilation. The Petri dishes were then placed in a climate chamber set at 25 ± 2 °C and 75% humidity (Castañeda-Ruiz et al. 2016). The samples were observed every three days for the formation of mycelium or fruiting bodies on the surface, and sterile water was added to maintain moisture. This observation continued for thirty days. Small amounts of mycelium or individual fruiting bodies were picked up with a loop and inoculated onto potato dextrose agar (PDA) plates, or the tip of the conidial pile was touched and streaked using the three-zone streaking method to isolate single colonies, thereby obtaining pure cultures. The cultures were incubated at 25 ± 2 °C for 1–2 weeks. The obtained isolates were stored in the Forest Pathology Laboratory at Nanjing Forestry University. Holotype specimens of new species from this study were deposited at the China Forestry Culture Collection Center (CFCC, https://cfcc.caf.ac.cn/), Chinese Academy of Forestry, Beijing, China.

**Table 1.** List of palm samples.

Latin Name	Origin	Sampling Time	Isolation Time
*Kentiopsis oliviformis*	New Caledonia	Nov. 8, 2024	Nov. 11, 2024
*Ptychosperma* sp.	New Guinea	Nov. 8, 2024	Nov. 13, 2024
*Washingtonia filifera*	United States, Mexico	Nov. 8, 2024	Nov. 15, 2024
*Latania lontaroides*	Mascarene Islands	Nov. 8, 2024	Nov. 15, 2024

### ﻿Morphological studies

Mycelial blocks measuring 5 mm in diameter were placed at the center of the culture medium and incubated in the dark at 25 ± 2 °C. After 7 days of incubation, colony characteristics were observed and recorded. Micromorphological structures were photographed using a Zeiss stereo microscope. Distilled water was used as the mounting medium. Morphological observations of reproductive structures were conducted using a Zeiss fluorescence microscope. The imaging software used was ZEN 3.9. For each structure, 30 measurements were made. Colors were determined using Kornerup and Wanscher (1978). Photo plates were prepared using Adobe Photoshop 2021.

### ﻿Molecular analyses

Fungal tissue was scraped from pure cultures and placed into a 2 mL centrifuge tube. A total of 600 µL of CTAB extraction buffer (2% CTAB, 1.4 M NaCl, 20 mM EDTA [pH 8.0], 100 mM Tris-HCl [pH 8.0], 0.2% β-mercaptoethanol) and 600 µL of chloroform were added. The mixture was vortexed and then incubated at room temperature for 4 hours with continuous shaking. The Eppendorf tube was centrifuged at high speed for 10 minutes at 4 °C. The supernatant was transferred to a 1.5 mL centrifuge tube. An equal volume of cold isopropanol was added to precipitate the DNA, followed by centrifugation at high speed for 5 minutes. The supernatant was carefully removed, and the DNA pellet was washed with 600 µL of 75% ethanol. After centrifugation for 5 minutes, the ethanol was removed, and the DNA pellet was air-dried. The DNA was dissolved in 30 µL of double-distilled water and stored at −20 °C. The quality of the extracted DNA was assessed using a microvolume spectrophotometer.

The rDNA region of ITS1-5.8S-ITS2 (internal transcribed spacer, ITS) was amplified using the primer pair ITS1 and ITS4 (White et al. 1990). The 28S nrRNA gene (LSU) was amplified using the primer pair NL1/NL4 (Kurtzman and Robnett 1997). Actin (*ACT*) was amplified using the primers ACT-512F/ACT-783R (Carbone and Kohn 1999). The translation elongation factor 1-alpha (*TEF*) gene was amplified using the primers EF1-728F/EF1-986R (Carbone and Kohn 1999). PCR amplification was carried out in a 25 µL reaction mixture consisting of 1 µL template DNA, 12.5 µL Taq polymerase, 1 µL of each primer, and 9.5 µL double-distilled water. The PCR conditions were as follows: 95 °C for 3 min, followed by 35 cycles of 95 °C for 1 min, 50–55 °C for 1 min, and 72 °C for 1 min. The final extension step was 72 °C for 10 min. The primers were synthesized by Sangon Biotech, Nanjing, Jiangsu Province, China. The amplified products were sequenced at Shanghai Jie Li Biotechnology Co., Ltd. The primer sequences are shown in Table 2.

**Table 2.** PCR primers and annealing temperature.

Gene	Primer	5’-Sequence-3’	Reference	Annealing temperature
ITS	ITS1	TCCGTAGGTGAACCTGCGG	White et al. 1990	55 °C
	ITS4	TCCTCCGCTTATTGATATGC		
LSU	NL1	GCATATCAATAAGCGGAGGAAAAG	Kurtzman and Robnett 1997	55 °C
	NL4	GGTCCGTGTTTCAAGACGG		
ACT	ACT-512F	ATGTGCAAGGCCGGTTTCGC	Carbone and Kohn 1999	54 °C
	ACT-783R	TACGAGTCCTTCTGGCCCAT		
TEF	EF1-728F	CATCGAGAAGTTCGAGAAGG	Carbone and Kohn 1999	54 °C
	EF1-986R	TACTTGAAGGAACCCTTACC		

To include a more comprehensive range of species, gene regions were selected based on previous research: ITS + *ACT* + *TEF* for *Cladosporium* and ITS + LSU for *Sarocladium* (Lee et al. 2023; Ou et al. 2020). The dataset was then aligned separately using MAFFT v.7.4 (http://mafft.cbrc.jp/alignment/server/) (Katoh and Standley 2013) with the G-INS-i iterative refinement algorithm and optimized manually in BioEdit v.7.0.5.3 (Tables 3, 4) (Hall 1999). The separate alignments were concatenated using PhyloSuite v.1.2.2 (Zhang et al. 2020).

**Table 3.** List of *Cladosporium* strains with their current names, origin, and GenBank accession numbers. New sequences are in bold; “–” represents missing data. * indicates ex-type strain.

Species complex	Species	Culture	Origin	GenBank Accession Number		
				ITS	ACT	TEF
* cladosporioides *	*C. angustiterminale*	CBS 140480*	*Banksia grandis*, Australia	KT600379	KT600575	KT600476
* cladosporioides *	*C. anthropophilum*	CBS 140685*	Man, bronchoalveolar lavage fluid, USA: MN	LN834437	LN834621	LN834533
* cladosporioides *	*C. asperulatum*	CBS 126340*	*Protea susannae*, Portugal	HM147998	HM148485	HM148239
* cladosporioides *	*C. australiense*	CBS 125984*	*Eucalyptus moluccana*, Australia	HM147999	HM148486	HM148240
* cladosporioides *	*C. cavernicola*	URM 8389*	Air, cave, Brazil	MZ518829	MZ555746	MZ555733
* cladosporioides *	*C. chalastosporoides*	CBS 125985*	*Teratosphaeria proteae-arboreae* on leaves of *Protea nitida*, South Africa	HM148001	HM148488	HM148242
* cladosporioides *	*C. chasmanthicola*	CBS 142612*	*Chasmanthe aethiopica*, leaf spots, South Africa	KY646221	KY646224	KY646227
* cladosporioides *	*C. chubutense*	CBS 124457*	*Pinus ponderosa*, Argentina	FJ936158	FJ936165	FJ936161
* cladosporioides *	*C. delicatulum*	CBS 126344*	*Tilia cordata*, leaves, Germany	HM148081	HM148570	HM148325
* cladosporioides *	*C. devikae*	BRIP 72278a*	*Macadamia integrifolia*, flower blight, Australia	MZ303808	MZ344212	MZ344193
* cladosporioides *	*C. endoviticola*	JZB390018*	*Vitis vinifera*, flower, China	MN654960	MN984220	MN984228
* cladosporioides *	*C. exile*	CBS 125987*	Chasmothecia of *Phyllactinia guttata* on leaves of *Corylus avellana*, USA: WA	HM148091	HM148580	HM148335
* cladosporioides *	*C. flabelliforme*	CBS 126345*	*Melaleuca cajuputi*, Australia	HM148092	HM148581	HM148336
* cladosporioides *	*C. flavovirens*	CBS 140462*	Man, toenail, USA: FL	LN834440	LN834624	LN834536
* cladosporioides *	*C. funiculosum*	CBS 122129*	*Vigna umbellata*, Japan	HM148094	HM148583	HM148338
* cladosporioides *	*C. fuscoviride*	FMR 16385*	Garden soil, Spain	LR813200	LR813206	LR813212
* cladosporioides *	*C. grevilleae*	CBS 114271*	*Grevillea* sp., leaves, Australia	JF770450	JF770473	JF770472
* cladosporioides *	*C. hillianum*	CBS 125988*	*Typha orientalis*, leaf mold, New Zealand	HM148097	HM148586	HM148341
* cladosporioides *	*C. inversicolor*	CBS 401.80*	*Triticum aestivum*, leaf, Netherlands	HM148101	HM148590	HM148345
* cladosporioides *	*C. ipereniae*	CBS 140483*	*Puya* sp., Chile	KT600394	KT600589	KT600491
* cladosporioides *	*C. iranicum*	CBS 126346*	*Citrus sinensis*, leaf, Iran	HM148110	HM148599	HM148354
* cladosporioides *	*C. lagenariiforme*	SFC20230103-M23*	Seaweed	OQ186119	OQ185167	OQ185128
* cladosporioides *	*C. licheniphilum*	CBS 125990*	Lichen *Phaeophysica orbicularis* and *Physcia* sp. on stems and bark of *Acer platanoides*, Germany	HM148111	HM148600	HM148355
* cladosporioides *	*C. longicatenatum*	CBS 140485*	Unknown plant, Australia	KT600403	KT600598	KT600500
* cladosporioides *	*C. lycoperdinum*	CBS 574.78C	*Aureobasidium caulivorum*, Russia	HM148115	HM148604	HM148359
* cladosporioides *	*C. neopsychrotolerans*	CGMCC 3.18031*	*Saussurea involucrata*, rhizosphere soil, China	KX938383	KX938366	KX938400
* cladosporioides *	*C. oxysporum*	CBS 125991	Soil, near the terracotta army, China	HM148118	HM148607	HM148362
* cladosporioides *	*C. oxysporum*	CBS 126351	Indoor air, Venezuela	HM148119	HM148608	HM148363
* cladosporioides *	*C. paracladosporioides*	CBS 171.54*	—	HM148120	HM148609	HM148364
* cladosporioides *	*C. parapenidielloides*	CBS 140487*	*Eucalyptus* sp., Australia	KT600410	KT600606	KT600508
* cladosporioides *	*C. passiflorae*	COAD 2135*	*Passiflora edulis*, leaf, Brazil	MH682175	MH729795	MH724943
* cladosporioides *	*C. phaenocomae*	CBS 128769*	*Phaenocoma prolifera*, South Africa	JF499837	JF499881	JF499875
* cladosporioides *	*C. phyllactiniicola*	CBS 126352*	Chasmothecia of *Phyllactinia guttata* on leaves of *Corylus avellana*, USA: WA	HM148150	HM148639	HM148394
* cladosporioides *	*C. phyllophilum*	CBS 125992*	*Taphrina* sp. on *Prunus cerasus*, Germany	HM148154	HM148643	HM148398
* cladosporioides *	*C. pini-ponderosae*	CBS 124456*	*Pinus ponderosa*, Argentina	FJ936160	FJ936167	FJ936164
* cladosporioides *	*C. proteacearum*	BRIP 72301a*	*Macadamia integrifolia*, flower blight, Australia	MZ303809	MZ344213	MZ344194
* cladosporioides *	*C. puris*	COAD 2494	Submerged litter in streams, Brazil	MK253338	MK249981	MK293778
* cladosporioides *	*C. rubrum*	CMG 28*	*Ulva* sp. (synonym: *Enteromorpha* sp.), Portugal	MN053018	MN066639	MN066644
* cladosporioides *	*C. rugulovarians*	CBS 140495*	Unidentified Poaceae, leaf sheaths, Brazil	KT600459	KT600656	KT600558
* cladosporioides *	*C. scabrellum*	CBS 126358*	*Ruscus hypoglossum*, Slovenia	HM148195	HM148685	HM148440
* cladosporioides *	*C. silenes*	CBS 109082*	*Silene maritima*, UK: Wales	EF679354	EF679506	EF679429
* cladosporioides *	*C. sinuatum*	CGMCC 3.18096*	Alpine Soil, China	KX938385	KX938368	KX938402
* cladosporioides *	*C. subuliforme*	CBS 126500*	*Chamaedorea metallica*, Thailand	HM148196	HM148686	HM148441
* cladosporioides *	*C. tenuissimum*	CPC 22398	Air, classroom, USA: TX	MF473285	MF474135	MF473708
* cladosporioides *	*C. tianshanense*	CGMCC 3.18033*	*Saussurea involucrata*, rhizosphere soil, China	KX938381	KX938364	KX938398
* cladosporioides *	*C. uredinicola*	CPC 5390	Hyperparasite on Cronartium fusiforme f. sp. quercum on *Quercus nigra* leaves, USA: AL	AY251071	HM148712	HM148467
* cladosporioides *	*C. uwebraunianum*	CBS 143365*	Indoor air, archive, Netherlands	MF473306	MF474156	MF473729
* cladosporioides *	*C. varians*	CBS 126362*	*Catalpa bungei*, Russia	HM148224	HM148715	HM148470
* cladosporioides *	*C. verrucocladosporioides*	CBS 126363*	*Rhus chinensis*, South Korea	HM148226	HM148717	HM148472
* cladosporioides *	*C. welwitschiicola*	CBS 142614*	*Welwitschia mirabilis*, dead leaf, Namibia	KY646223	KY646226	KY646229
* cladosporioides *	*C. westerdijkiae*	CBS 113746*	Bing cherry fruits, USA: WA	HM148061	HM148548	HM148303
* cladosporioides *	*C. xanthochromaticum*	CBS 140691*	Man, bronchoalveolar lavage fluid, USA: TX	LN834415	LN834599	LN834511
* cladosporioides *	*C. xanthochromaticum*	DTO 323-E2	Indoor air, China	MF473319	MF474169	MF473742
* cladosporioides *	*C. xylophilum*	CBS 125997*	*Picea abies*, dead wood, Russia	HM148230	HM148721	HM148476
* herbarum *	*C. aerium*	CBS 143356*	Indoor air, China	MF472897	MF473747	MF473324
* herbarum *	*C. aggregatocicatricatum*	CBS 140493*	Culture contaminant, New Zealand	KT600448	KT600645	KT600547
* herbarum *	*C. allicinum*	CBS 121624*	*Hordeum vulgare*, Belgium	EF679350	EF679502	EF679425
* herbarum *	*C. antarcticum*	CBS 690.92*	*Caloplaca regalis*, Antarctica	EF679334	EF679484	EF679405
* herbarum *	*C. arthropodii*	CBS 124043**	*Arthropodium cirratum*, New Zealand	JN906979	JN906998	JN906985
* herbarum *	*C. fildesense*	F09-T12-1*	An unidentified marine sponge, Antarctica	JX845290	MN233632	MN233633
* herbarum *	*C. floccosum*	CBS 140463*	Man, ethmoid sinus, USA: MN	LN834416	LN834600	LN834512
* herbarum *	*C. herbaroides*	CBS 121626*	Hypersaline water, salterns, Israel	EF679357	EF679509	EF679432
* herbarum *	*C. herbarum*	CBS 121621**	*Hordeum vulgare*, Netherlands	EF679363	EF679516	EF679440
* herbarum *	*C. iridis*	CBS 138.40**	*Iris* sp., leaves, Netherlands	EF679370	EF679523	EF679447
* herbarum *	*C. limoniforme*	CBS 140484*	*Musa acuminata*, Egypt	KT600397	KT600592	KT600494
* herbarum *	*C. macrocarpum*	CBS 121623*	*Spinacia oleracea*, USA: WA	EF679375	EF679529	EF679453
* herbarum *	*C. ossifragi*	CBS 842.91*	*Narthecium ossifragum*, green leaf, Norway	EF679381	EF679535	EF679459
* herbarum *	*C. paralimoniforme*	CGMCC 3.18103*	Meadow soil, China	KX938392	KX938375	KX938409
* herbarum *	*C. phlei*	CBS 358.69**	*Phleum pratense*, Germany	JN906981	JN907000	JN906991
* herbarum *	*C. prolongatum*	CGMCC 3.18036*	*Populus euphratica*, rhizosphere soil, China	KX938394	KX938377	KX938411
* herbarum *	*C. pseudiridis*	CBS 116463*	*Iris* sp., large leaf lesions, New Zealand	EF679383	EF679537	EF679461
* herbarum *	*C. pseudotenellum*	FMR 16231*	Garden soil, Spain	LR813145	LR813146	LR813196
* herbarum *	*C. puyae*	CBS 274.80A*	*Puya goudotiana*, Colombia	KT600418	KT600614	KT600516
* herbarum *	*C. ramotenellum*	CBS 121628*	Hypersaline water, Slovenia	EF679384	EF679538	EF679462
* herbarum *	*C. rhusicola*	CBS 140492*	*Rhus* sp., South Africa	KT600440	KT600637	KT600539
* herbarum *	*C. sinense*	CBS 143363*	Indoor air, China	MF473252	MF474102	MF473675
* herbarum *	*C. sinuosum*	CBS 121629*	*Fuchsia excorticata*, New Zealand	EF679386	EF679540	EF679464
* herbarum *	*C. soldanellae*	CBS 132186*	*Soldanella alpina*, Germany	JN906982	JN907001	JN906994
* herbarum *	*C. spinulosum*	CBS 119907*	Hypersaline water, Slovenia	EF679388	EF679542	EF679466
* herbarum *	*C. subcinereum*	CBS 140465*	Man, sputum, USA: MT	LN834433	LN834617	LN834529
* herbarum *	*C. subinflatum*	CBS 121630*	Hypersaline water, saltern, Slovenia	EF679389	EF679543	EF679467
* herbarum *	*C. submersum*	FMR 17264*	Submerged plant material, Spain	LR813144	LR813195	LR813197
* herbarum *	*C. variabile*	CBS 121635**	*Spinacia oleracea*, USA: WA	EF679403	EF679557	EF679481
* herbarum *	*C. verruculosum*	CGMCC 3.18099*	Alpine soil, China	KX938388	KX938371	KX938405
* herbarum *	*C. versiforme*	CBS 140491*	*Hordeum* sp., Iran	KT600417	KT600613	KT600515
* herbarum *	*C. wyomingense*	CBS 143367*	Indoor air, living room, USA: WY	MF473315	MF474165	MF473738
* sphaerospermum *	*C. aciculare*	CBS 140488*	*Syzygium corynanthum*, Australia	KT600411	KT600607	KT600509
* sphaerospermum *	*C. aphidis*	CBS 132182**	Unidentified aphid, Germany	JN906978	JN906997	JN906984
* sphaerospermum *	*C. austrohemisphaericum*	CBS 140482*	*Lagunaria patersonia*, black mold on fruit surface, New Zealand	KT600382	KT600578	KT600479
* sphaerospermum *	*C. coloradense*	CBS 143357*	Air sample, bedroom, USA: CO	MF472945	MF473795	MF473372
* sphaerospermum *	*C. cycadicola*	CBS 137970*	*Cycas media*, leaves, Australia	KJ869122	KJ869227	KJ869236
* sphaerospermum *	*C. domesticum*	CBS 143358*	Indoor air, USA: NJ	MF472955	MF473805	MF473382
* sphaerospermum *	*C. domesticum*	CPC 22402	Indoor air, classroom, USA: TX	MF472959	MF473809	MF473386
* sphaerospermum *	*C. dominicanum*	CBS 119415*	Hypersaline water, saltern, Dominican Republic	DQ780353	KJ596641	JN906986
* sphaerospermum *	*C. fusiforme*	CBS 119414*	Hypersaline water, saltern, Slovenia	DQ780388	KJ596640	JN906988
* sphaerospermum *	*C. fusiforme*	EXF-397	Hypersaline water, saltern, Slovenia	DQ780389	EF101373	KJ596595
* sphaerospermum *	*C. halotolerans*	CBS 119416*	Hypersaline water, salterns, Namibia	DQ780364	KJ596633	JN906989
* sphaerospermum *	*C. halotolerans*	CPC 22335	Air, bedroom, USA: NJ	MF472992	MF473841	MF473420
* sphaerospermum *	*C. halotolerans*	SFC20230103-M06	Sea water, OMZ	OQ165238	OQ184989	—
* sphaerospermum *	*C. halotolerans*	SFC20230103-M07	Sea water, OMZ	OQ165239	OQ184990	OQ185117
* sphaerospermum *	*C. langeronii*	CBS 189.54*	Man, mycosis, Brazil	DQ780379	EF101357	JN906990
* sphaerospermum *	*C. lebrasiae*	CBS 138283*	Milk bread, France	KJ596568	KJ596631	KJ596583
* sphaerospermum *	*C. longissimum*	CBS 300.96*	Soil along coral reef coast, Papua New Guinea	DQ780352	EF101385	EU570259
* sphaerospermum *	*C. marinisedimentum*	MABIK FU00001143	Deep-sea sediment	OQ186123	OQ185171	OQ185132
* sphaerospermum *	*C. marinisedimentum*	SFC20230103-M28*	Deep-sea sediment	OQ186124	OQ185172	OQ185133
* sphaerospermum *	*C. neolangeronii*	CBS 109868	Mortar of Muro Farnesiano, Italy	DQ780377	EF101362	MF473571
* sphaerospermum *	*C. neolangeronii*	CBS 797.97*	Indoor environment, Netherlands	MF473143	MF473992	—
* sphaerospermum *	*C. parahalotolerans*	CBS 139585*	Swab sample, apartment, Netherlands	KP701955	KP702077	KP701832
* sphaerospermum *	*C. parahalotolerans*	DTO 324-B7	Indoor air, China	MF473169	MF474017	MF473592
* sphaerospermum *	*C. penidielloides*	CBS 140489*	*Acacia verticillata*, Australia	KT600412	KT600608	KT600510
* sphaerospermum *	*C. psychrotolerans*	CBS 119412*	Hypersaline water, Slovenia	DQ780386	KJ596632	JN906992
* sphaerospermum *	*C. pulvericola*	CBS 143362*	House dust, New Zealand	MF473226	MF474075	MF473648
* sphaerospermum *	*C. ruguloflabelliforme*	CBS 140494*	Diatrapaceae sp. on *Aloe* sp., South Africa	KT600458	KT600655	KT600557
* sphaerospermum *	*C. salinae*	CBS 119413*	Hypersaline water, saltern, Slovenia	DQ780374	EF101390	JN906993
* sphaerospermum *	*C. sloanii*	CBS 143364*	Swab sample, food plant, Netherlands	MF473253	MF474103	MF473676
* sphaerospermum *	*C. sphaerospermum*	CBS 139576	Indoor environment, Germany	KP701884	KP702007	KP701761
* sphaerospermum *	*C. sphaerospermum*	CBS 193.54*	Man, nails, Netherlands	DQ780343	EU570269	EU570261
* sphaerospermum *	*C. succulentum*	CBS 140466*	Dolphin, bronchus, USA: FL	LN834434	LN834618	LN834530
* sphaerospermum *	*C. velox*	CBS 119417*	*Bambusa* sp., India	DQ780361	EF101388	JN906995
* sphaerospermum *	*C. velox*	CPC 22359	Indoor air sample, USA: MA	MF473308	MF474158	MF473731
* sphaerospermum *	***C. menglunense* sp. nov.**	Psp2-7a1*	***Ptychosperma* sp., dead leaves, Yunnan, China**	**PV737441**	**PV759127**	**PV759135**
* sphaerospermum *	***C. menglunense* sp. nov.**	**Psp2-7a2**	***Ptychosperma* sp., dead leaves, Yunnan, China**	**PV737442**	**PV759128**	**PV759136**
* sphaerospermum *	***C. menglunense* sp. nov.**	**Psp2-7a3**	***Ptychosperma* sp., dead leaves, Yunnan, China**	**PV737443**	**PV759129**	**PV759137**
* sphaerospermum *	***C. menglunense* sp. nov.**	**Psp2-7a4**	***Ptychosperma* sp., dead leaves, Yunnan, China**	**PV737444**	**PV759130**	**PV759138**
Outgroup	*Cercospora beticola*	CBS 116456	*Beta vulgaris*, Italy	AY840527	AY840458	AY840494

**Table 4.** List of *Sarocladium* strains with their current names, origin, and GenBank accession numbers. New sequences are in bold; “–” represents missing data. * indicates ex-type strain.

Species	Culture	Origin	GenBank Accession Number	
			ITS	LSU
*S. bacillisporum*	CBS 425.67*	Soil, Ontario, Canada	HE608639	MH870718
*S. bactrocephalum*	CBS 749.69*	*Ustilago* sp., Canada	HG965006	HQ231994
*S. bifurcatum*	UTHSC 05-3311*	Bronchoalveolar lavage fluid, USA	HG965009	HG965057
*S. brachiariae*	CGMCC 2192*	Leaves of *Brachiaria brizantha*, China	EU880834	KP715271
*S. dejongiae*	CBS 144929*	Soil, The Netherlands	MK069419	MK069415
*S. gamsii*	CBS 707.73*	Dead stem of *Pandanus lerum*, Sri Lanka	HG965015	HG965063
*S. glaucum*	CBS 796.69*	Woolen overcoat, Solomon Islands	OQ429841	HE608657
*S. hominis*	UTHSC 04-1034*	Right calf tissue, USA	HG965012	HG965060
*S. hominis*	UTHSC 02-2564	Leg, USA	HG965011	—
*S. hominis*	UTHSC 04-3464	Sputum, USA	HG965013	HG965061
*S. implicatum*	CBS 959.72*	Desert soil, Egypt	HG965023	HG965072
*S. kiliense*	CBS 122.29*	Skin, Germany	AJ621775	HQ232052
*S. ochraceum*	CBS 428.67*	*Zea mays*, Kenya	HG965025	HQ232070
*S. oryzae*	CBS 180.74*	*Oryza sativa*, India	HG965026	HG965047
*S. oryzae*	CBS 399.73	*Oryza sativa*, India	HG965027	HG965048
*S. oryzae*	CBS 414.81	*Oryza sativa*, Nigeria	HG965028	HG965049
*S. pseudostrictum*	UTHSC 02-1892*	Sputum, USA	HG965029	HG965073
*S. spinificis*	Z0106 Ex-type	Root of *Spinifex littoreus*, Taiwan, ROC	KF269096	JQ954463
*S. strictum*	CBS 346.70*	*Triticum aestivum*, Germany	FN691453	HQ232141
*S. subulatum*	MUCL 9939*	Soil, Egypt	HG965031	HG965075
*S. summerbellii*	CBS 430.70*	Soil from greenhouse, The Netherlands	HG965034	HG965078
*S. terricola*	CBS 243.59*	Forest soil, USA	MH857853	MH869389
*S. theobromae*	CBS 113440 Ex-type	*Theobroma gileri*	OQ429856	OQ430108
*S. zeae*	CBS 800.69*	*Zea mays* stalk, USA	FN691451	HQ232152
***S. menglaense* sp. nov.**	KO1-8*	***Kentiopsis oliviformis*, dead leaves, Yunnan, China**	**PV737445**	**PV737452**
***S. menglaense* sp. nov.**	**LLon2-5A**	***Latania lontaroides*, dead leaves, Yunnan, China**	**PV737446**	**PV737453**
***S. menglaense* sp. nov.**	**LLon2-8a**	***Latania lontaroides*, dead leaves, Yunnan, China**	**PV737447**	**PV737454**
***S. menglaense* sp. nov.**	**LLon4-1c**	***Latania lontaroides*, dead leaves, Yunnan, China**	**PV737448**	**PV737455**
***S. yunnanense* sp. nov.**	WF8-2a1*	***Washingtonia filifera*, dead leaves, Yunnan, China**	**PV737449**	**PV737456**
***S. yunnanense* sp. nov.**	**WF8-2a2**	***Washingtonia filifera*, dead leaves, Yunnan, China**	**PV737450**	**PV737457**
***S. yunnanense* sp. nov.**	**WF8-2a3**	***Washingtonia filifera*, dead leaves, Yunnan, China**	**PV737451**	**PV737458**
*Chlamydocillium curvulum*	CBS 430.66*	Wheat field soil, Germany	HE608638	HE608656

Maximum likelihood (ML) analyses and Bayesian inference (BI) were carried out using RAxML v.8.2.10 (Stamatakis 2014) and MrBayes v.3.2.6 (Ronquist et al. 2012), respectively. In ML analysis, statistical support values were obtained using rapid bootstrapping with 1000 replicates, with default settings for other parameters. For BI, the best-fit partitioning scheme and substitution model were determined using ModelFinder (Kalyaanamoorthy et al. 2017) via the “greedy” algorithm, and branch lengths were estimated as “linked” and AICc. Four Markov chain Monte Carlo chains (one cold) were constructed for 5,000,000 generations, with sampling every 1000 generations. Convergence was assessed as a standard deviation of split frequencies < 0.01. The first quarter of the trees, which represented the burn-in phase of the analyses, was discarded, and the remaining trees were used to calculate posterior probabilities (BPP) in the majority rule consensus tree.

Phylogenetic trees were visualized using FigTree v.1.4.4 (Rambaut 2018). Branches that received bootstrap support in ML (≥ 75%) and BPP (≥ 0.95) were considered significantly supported.

## ﻿Results

### ﻿Phylogeny

**Figure 1.**
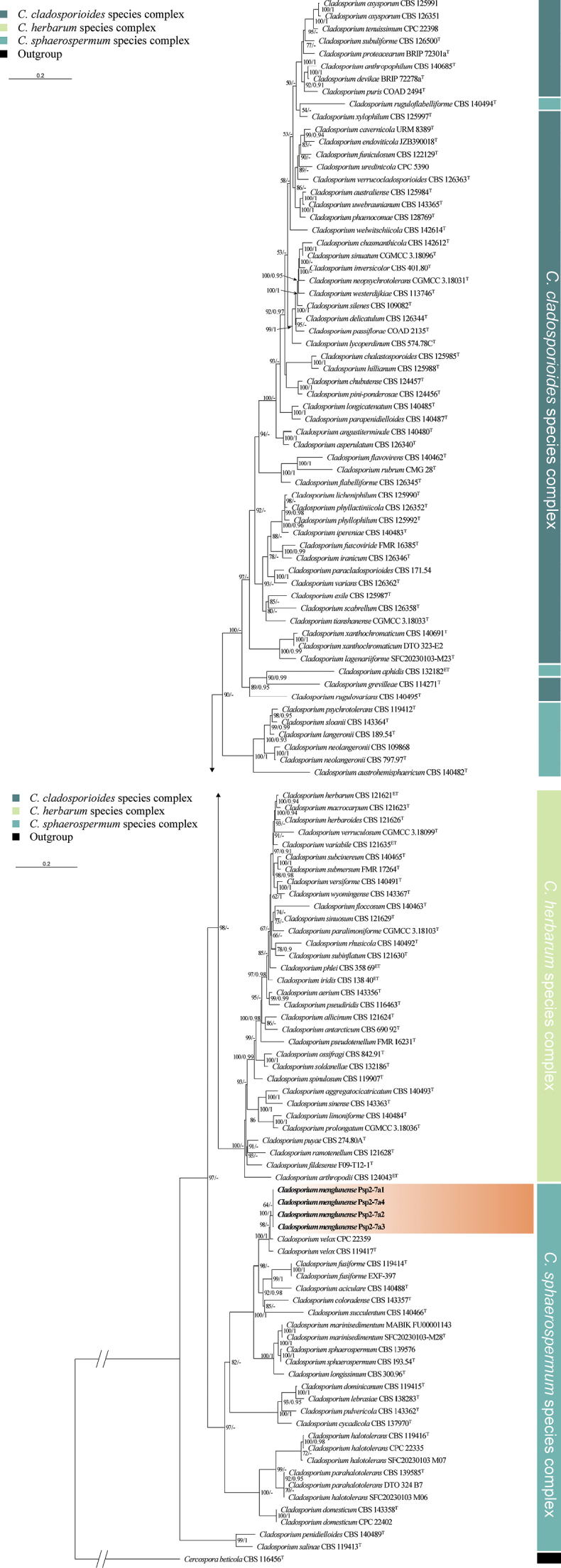
Phylogram of *Cladosporium* resulting from a maximum likelihood analysis based on the ITS + *ACT* + *TEF* sequence. Numbers above the branches indicate ML bootstrap values (left, ML BS ≥ 50%) and Bayesian posterior probabilities (right, BPP ≥ 0.90). T = ex-type strain.

The *Cladosporium* dataset comprised three genes (ITS + *ACT* + *TEF*) from 125 specimens representing 115 species. Sequences of *Cercospora beticola* Sacc. were retrieved from GenBank and used as the outgroup, as in Lee et al. (2023). The dataset had an aligned length of 1156 characters, including 234 sequences of ITS, 516 of *ACT*, and 404 of *TEF*. The best model for the *Cladosporium* dataset estimated and applied in the Bayesian analysis was GTR+F+I+G4, with an average standard deviation of split frequencies = 0.071342 (BI). In our phylogenetic tree (Fig. 1), most *Cladosporium* species clustered within three groups, which correspond to three complexes, viz. *C. cladosporioides*, *C. herbarum*, and *C. sphaerospermum*. Additionally, four strains (Psp 2-7a1, Psp 2-7a2, Psp 2-7a3, and Psp 2-7a4), representing a new species, clustered in the *C. sphaerospermum* complex, forming an independent lineage with strong support (100/1.00), related to *C. velox* Zalar, de Hoog & Gunde-Cim.

**Figure 2.**
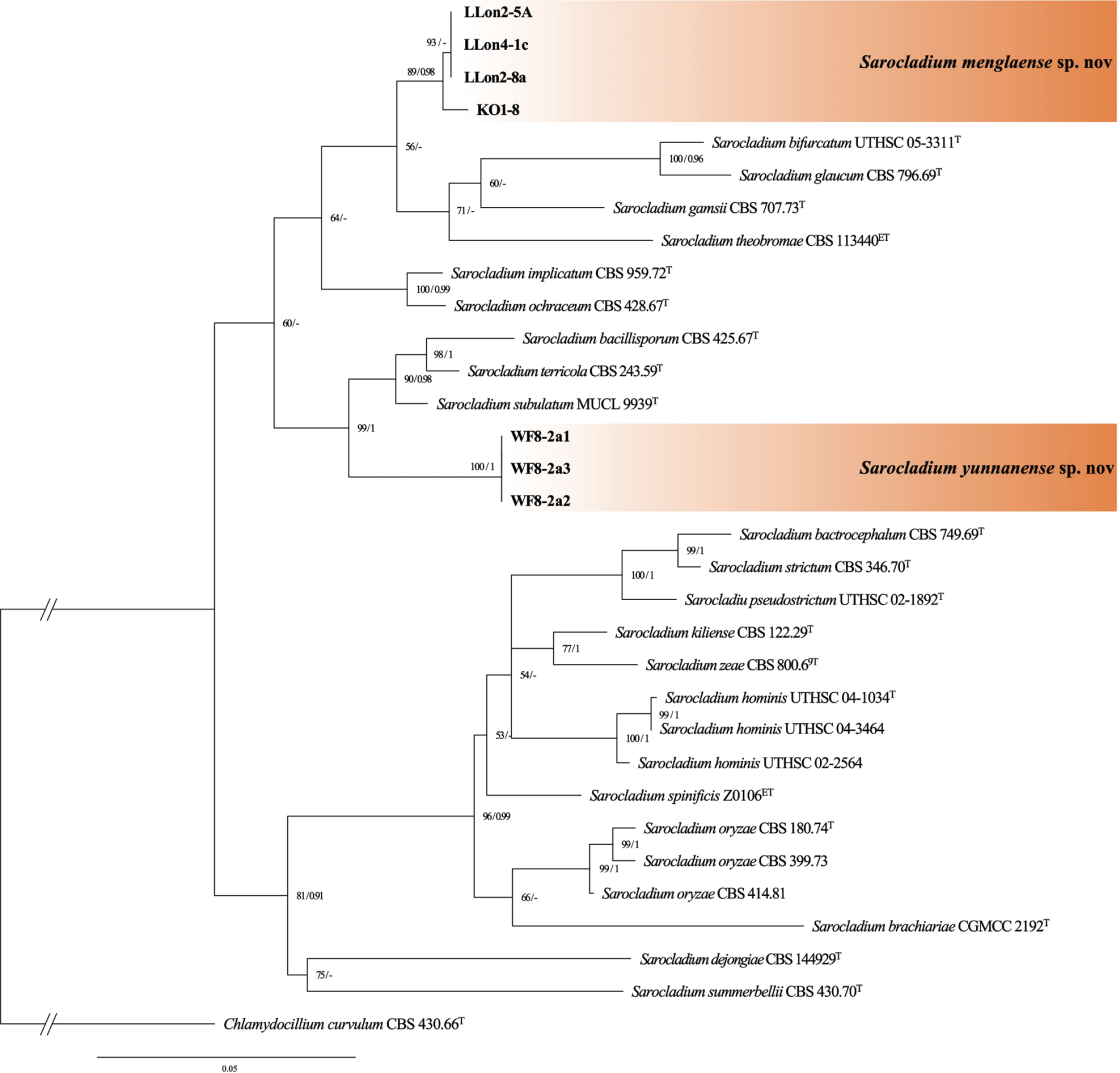
Phylogram of *Sarocladium* resulting from a maximum likelihood analysis based on the ITS + LSU sequence. Numbers above the branches indicate ML bootstrap values (left, ML BS ≥ 50%) and Bayesian posterior probabilities (right, BPP ≥ 0.90). T = ex-type strain.

The *Sarocladium* dataset comprised two genes (ITS + LSU) from 32 specimens representing 24 species. Sequences of *Chlamydocillium curvulum* (W. Gams) L.W. Hou, L. Cai & Crous were retrieved from GenBank and used as the outgroup, as in Ou et al. (2020). The dataset had an aligned length of 1075 characters, including 533 sequences of ITS and 541 of LSU. The best model for the *Sarocladium* dataset estimated and applied in the Bayesian analysis was GTR+F+I+G4, with an average standard deviation of split frequencies = 0.008654 (BI). The phylogeny inferred from the ITS + LSU sequences (Fig. 2) showed that four strains (KO1-8, LLon2-5A, LLon2-8a, and LLon4-1c), representing a new species, formed a distinct lineage related to *S. bifurcatum* A. Giraldo, Gené & Deanna A. Sutton, *S. glaucum* (W. Gams) Summerb., *S. gamsii* A. Giraldo, Gené & Guarro, and *S. theobromae* L.W. Hou, L. Cai & Crous, but with weak support (56/–). In contrast, three strains (WF8-2a1, WF8-2a2, and WF8-2a3) formed a distinct lineage, grouping with *S. subulatum* A. Giraldo, Gené & Guarro, *S. terricola* (J.H. Mill., Giddens & A.A. Foster) A. Giraldo, Gené & Guarro, and *S. bacillisporum* (Onions & G.L. Barron) Summerb., with strong support (99/1.00).

### ﻿Taxonomy

#### ﻿ Cladosporium menglunense

Taxon classificationFungiCladosporialesCladosporiaceae

L.H. Zhu, Lin Zhou & D.W. Li sp. nov.

6E3CDD1F-9A8E-5A8B-816D-3067BB6C0FBB

860644

Fig. 3

##### Type.

China • Yunnan Province, Xishuangbanna Prefecture, Mengla County, Menglun Town, Xishuangbanna Tropical Botanical Garden (21°55'0.12"N, 101°15'E), isolated from dead leaves of *Ptychosperma* sp., Nov. 2024, L.H. Zhu & D.W. Li. Holotype specimen CFCC72680 is a living specimen being maintained via lyophilization at the China Forestry Culture Collection Center (CFCC). Ex-type (=Psp 2-7a2) is maintained at the Forest Pathology Laboratory, Nanjing Forestry University.

##### Etymology.

The term ‘*menglunense*’ indicates that this species was collected from Menglun Town.

##### Description.

Asexual morph on PDA: ***Mycelium*** immersed, composed of septate, branched, pale brown hyphae, 1.7–5.4 μm wide, local swelling, with the widest up to 6.5 μm. ***Conidiophores*** macronematous and micronematous, arising laterally or terminally from hyphae, septate, erect to slightly flexuous, non-nodulose, branched, up to 100 μm long, pale brown, verruculose. ***Conidiogenous cells*** integrated, terminal and intercalary, cylindrical to subcylindrical, bearing up to three protuberant, slightly darkened, and refractive conidiogenous loci. ***Ramoconidia*** 0(–2) septate, subcylindrical to ellipsoidal, sometimes calabash-like constricted at the center, (9.5–)9.9–21.8(–23.2) × (3.0–)3.0–4.3(–4.4) μm (mean ± SD = 14.5 ± 3.2 × 3.6 ± 0.4 um, n = 50), pale brown, smooth. ***Conidia*** forming branched chains, with up to 4 conidia in the terminal unbranched part, sometimes with a long neck between conidia, aseptate, pale brown, smooth to verruculose, with protuberant scars, slightly darkened, subglobose to globose, (2.9–)3.2–5.2(–5.2) × (2.6–)3.0–3.8(–4.2) μm (mean ± SD = 4.1 ± 0.6 × 3.3 ± 0.3 μm, n = 40). Sexual morph not observed.

**Figure 3.**
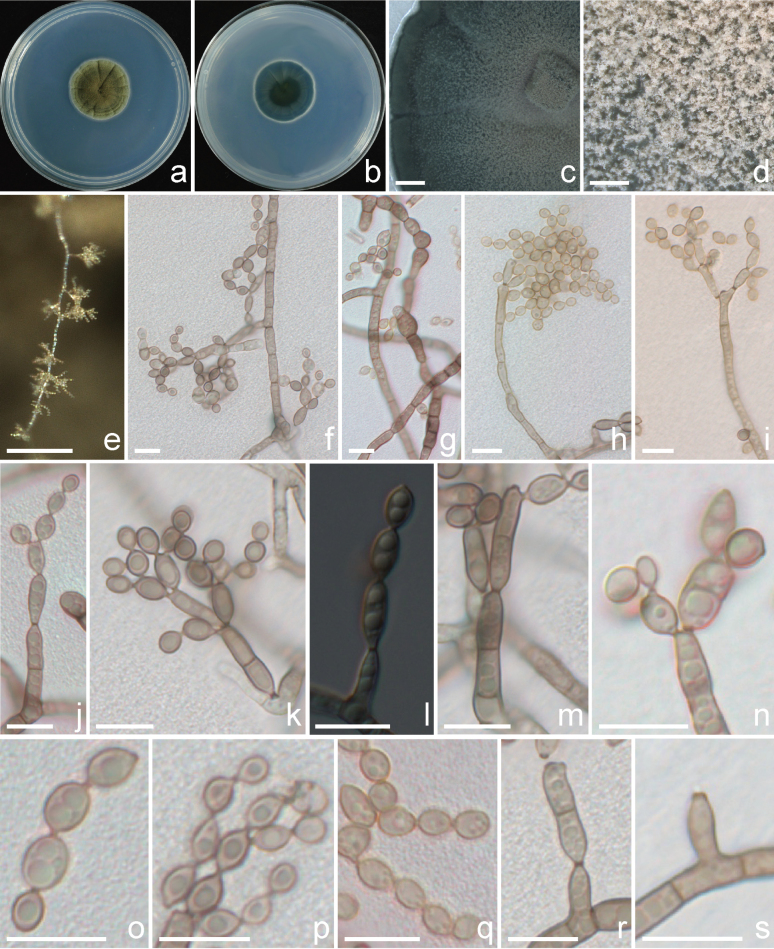
Morphology of *Cladosporium menglunense* (Psp 2-7a1 = CFCC72680). a, b. PDA culture colonies (a: front, b: reverse); c–e. The image observed under a stereomicroscope; f–k. Conidiophores and swollen hyphae; i. Early differentiating conidiophores; m, n. Ramoconidia and conidia; o–q. Conidial chains; r, s. Coronate scars on conidiogenous cells and ramoconidia. Scale bars: 2000 μm (c); 500 μm (d); 100 μm (e); 10 μm (f–s).

##### Cultural characters.

Colonies on PDA 2.4–3.1 cm diam after 7 days at 25 °C, yellowish-brown to olive, reverse atrovirens, floccose, velvety, crateriform, radially furrowed, wrinkled, usually significantly wrinkled at the margin; margin pale yellow edge; aerial mycelia abundantly formed, dense, with few exudates, powdery sporulation profuse.

##### Additional strains examined.

China • Yunnan Province, Xishuangbanna Prefecture, Mengla County, Menglun Town, Xishuangbanna Tropical Botanical Garden (21°55'0.12"N, 101°15'E), on dead leaves of *Ptychosperma* sp., Nov. 2024, L.H. Zhu & D.W. Li, Psp 2-7a3 (CFCC72682), Psp 2-7a4 (CFCC72683).

##### Notes.

Phylogenetically, *Cladosporium menglunense* nested in the *C. sphaerospermum* complex clade based on the ITS + *ACT* + *TEF* sequence data and was related to *C. velox* (Fig. 1). However, there was a 19 bp difference in the TEF gene fragment between C. menglunense and C. velox, with a similarity of 92.94%. Morphologically, *C. menglunense* can be easily distinguished from *C. velox* by its longer and wider conidia (3.2–5.2 × 3.0–3.8 μm vs. 2.5–4 × 2–2.5 μm). Furthermore, the ramoconidia of *C. menglunense* are smooth and can have up to 2 septa, whereas the ramoconidia of *C. velox* are either smooth or finely verrucose, with 0–1 septum. In addition, *C. velox* was isolated from *Bambusa* sp. (Poaceae), while *C. menglunense* was isolated from Arecaceae plants (Bensch et al. 2012).

#### ﻿ Sarocladium menglaense

Taxon classificationFungiCladosporialesCladosporiaceae

L.H. Zhu, Lin Zhou & D.W. Li sp. nov.

DF507389-2E9B-5D36-88BF-00FD4FFFFCBC

860645

Fig. 4

##### Type.

China • Yunnan Province, Xishuangbanna Prefecture, Mengla County, Menglun Town, Xishuangbanna Tropical Botanical Garden (21°55'0.12"N, 101°15'E), isolated from dead leaves of the *Kentiopsis oliviformis*, Nov. 2024, L.H. Zhu & D.W. Li. Holotype specimen CFCC72673 is a living specimen being maintained via lyophilization at the China Forestry Culture Collection Center (CFCC). Ex-type (=LLon2-5A) is maintained at the Forest Pathology Laboratory, Nanjing Forestry University.

##### Etymology.

The term ‘*menglaense*’ indicates that this species was collected from Mengla County.

##### Description.

Asexual morph on PDA: ***Mycelium*** immersed, composed of septate, branched, white to transparent hyphae, 1–2 μm wide. ***Conidiophores*** erect, arising directly from vegetative hyphae, simple, hyaline to subhyaline, (14.5–)18.1–56.2(–62.0) μm long (mean ± SD = 33.8 ± 9.1 μm, n = 60). ***Phialides*** solitary, straight or slightly flexuous, subulate, (14.5–)18.1–56.2(–62.0) μm long (mean ± SD = 33.8 ± 9.1 μm, n = 60), with distinct periclinal thickening of the conidiogenous loci, hyaline, thin- and smooth-walled. ***Conidia*** unicellular, cylindrical with rounded ends, (1.6–)2.3–5.8(–5.8) × (1.1–)1.2–2.1(–2.2) μm (mean ± SD = 3.4 ± 0.9 × 1.7 ± 0.2 μm, n = 60), hyaline to subhyaline, thin- and smooth-walled, arranged in slimy heads. Chlamydospores and sexual morph not observed.

**Figure 4.**
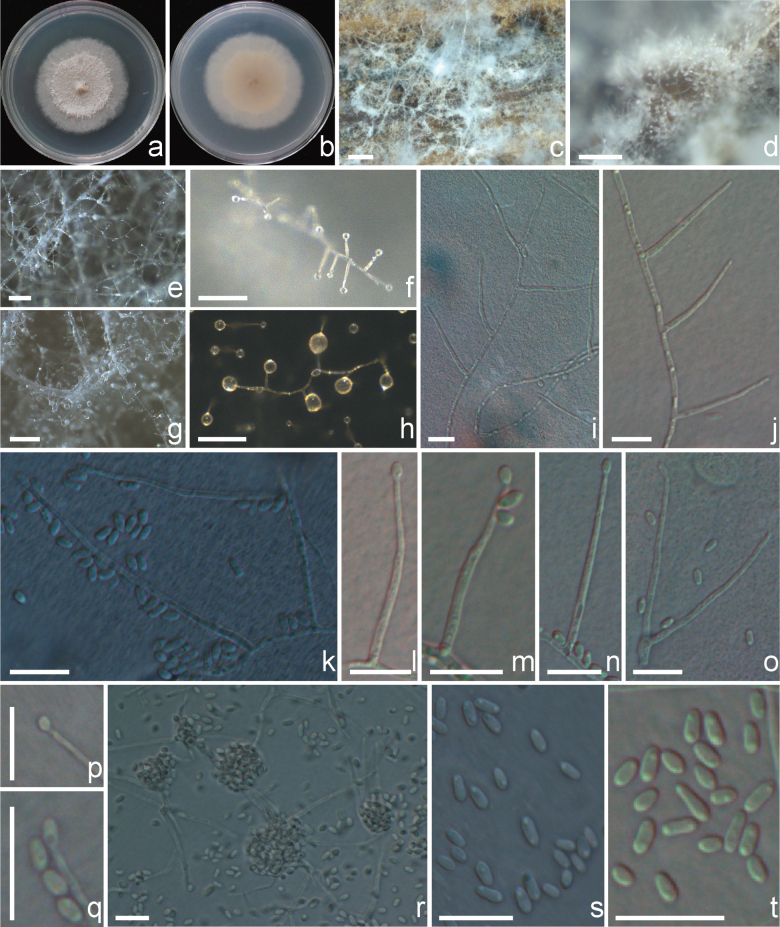
Morphology of *Sarocladium menglaense* (KO1-8 = CFCC72673). a, b. PDA culture colonies (a: front, b: reverse); c, d. Hyphae and sporulating structures on the host plant; e–h. Conidiogenous structures and spore masses on the colony; i, j. Conidiophores arranged on the hyphae; k–o. Conidiophores; p, q. Details of sporulation; r. Slimy head of spores; s, t. Conidia. Scale bars: 200 μm (c); 100 μm (d); 200 μm (e); 50 μm (f–g); 10 μm (h–t).

##### Cultural characters.

Colonies on PDA attaining 2.28–4.15 cm diam after 30 days at 25 °C, white to light gray, reverse yellowish white, flat, powdery; coarse flocculent in center, aerial mycelia occasionally seen near the edge; exudates not observed.

##### Additional strains examined.

China • Yunnan Province, Xishuangbanna Prefecture, Mengla County, Menglun Town, Xishuangbanna Tropical Botanical Garden (21°55'0.12"N, 101°15'E), dead leaves of *Latania lontaroides*, Nov. 2024, L.H. Zhu & D.W. Li, LLon2-8a (CFCC72675), and LLon4-1c (CFCC72676).

##### Notes.

Phylogenetically, *S. menglaense* nested in the *Sarocladium* clade based on the ITS + LSU sequence data, forming a distinct lineage. *S. menglaense* was related to *S. bifurcatum*, *S. glaucum*, *S. gamsii*, and *S. theobromae* with weak support (56/-) (Fig. 2). In terms of the ITS sequence, the similarities (bp difference) between *S. menglaense* and *S. bifurcatum*, *S. glaucum*, *S. gamsii*, and *S. theobromae* are 86.40% (76 bp difference), 87.18% (66 bp difference), 93.17% (37 bp difference), and 91.36% (42 bp difference), respectively. Whereas in the LSU, the similarities (bp difference) are 99.42% (3 bp difference), 98.65% (7 bp difference), 97.64% (13 bp difference), and 97.44% (14 bp difference), respectively. Morphologically, *S. menglaense* can be easily distinguished from *S. bifurcatum*, *S. gamsii*, and *S. theobromae* by its conidiophores being up to 62 μm long, versus up to 43 μm in *S. bifurcatum*, up to 55 μm in *S. gamsii* (Giraldo et al. 2015), and up to 112 μm in *S. theobromae* (Hou et al. 2023). In addition, *S. menglaense* can be clearly differentiated from *S. glaucum* by the color of the colony, which is white to transparent in the former but grey-green to bluish green in the latter (Gams 1971).

#### ﻿ Sarocladium yunnanense

Taxon classificationFungiCladosporialesCladosporiaceae

L.H. Zhu, Lin Zhou & D.W. Li sp. nov.

4200E6FE-D3CF-5F37-9795-BEB8B4A8323B

860646

Fig. 5

##### Type.

China • Yunnan Province, Xishuangbanna Prefecture, Mengla County, Menglun Town, Xishuangbanna Tropical Botanical Garden (21°55'0.12"N, 101°15'E), isolated from the dead leaves of the *Washingtonia filifera*, Nov. 2024, L.H. Zhu & D.W. Li. Holotype specimen CFCC72677 is a living specimen being maintained via lyophilization at the China Forestry Culture Collection Center (CFCC). Ex-type (=WF8-2a2) is maintained at the Forest Pathology Laboratory, Nanjing Forestry University.

##### Etymology.

The term ‘*yunnanense*’ indicates that this species was collected from Yunnan Province, China.

##### Description.

Asexual morph on PDA: ***Mycelium*** immersed, composed of septate, branched, white to transparent hyphae, 1.5–3 μm wide, with the aged mycelium chain-like. ***Conidiophores*** erect, arising directly from vegetative hyphae, simple, hyaline to subhyaline. ***Phialides*** solitary, straight or slightly flexuous, short needle-like, (11.3–)11.7–22.2(–25.2) μm (mean ± SD = 16.7 ± 3.4 μm, n = 20) long, with distinct periclinal thickening of the conidiogenous loci, hyaline, smooth, and thin-walled. ***Conidia*** unicellular, fusiform, (4.0–)4.2–7.0(–7.9) × (1.4–)1.4–2.0(–2.1) μm (mean ± SD = 4.8 ± 0.6 × 1.8 ± 0.2 μm, n = 50), with slightly truncate ends, initially hyaline and smooth, arranged in chains containing up to 15 spores; conidia become swollen and enlarged at a later stage, becoming subhyaline and apparently verruculose due to the production of a mucilaginous exudate, irregular or lemon-shaped with slightly truncate ends, (4.5–)4.5–6.8(–9.3) × (2.3–)2.3–3.7(–3.8) μm (mean ± SD = 5.6 ± 0.8 × 3.1 ± 0.4 μm, n = 50). Chlamydospores and sexual morph not observed.

**Figure 5.**
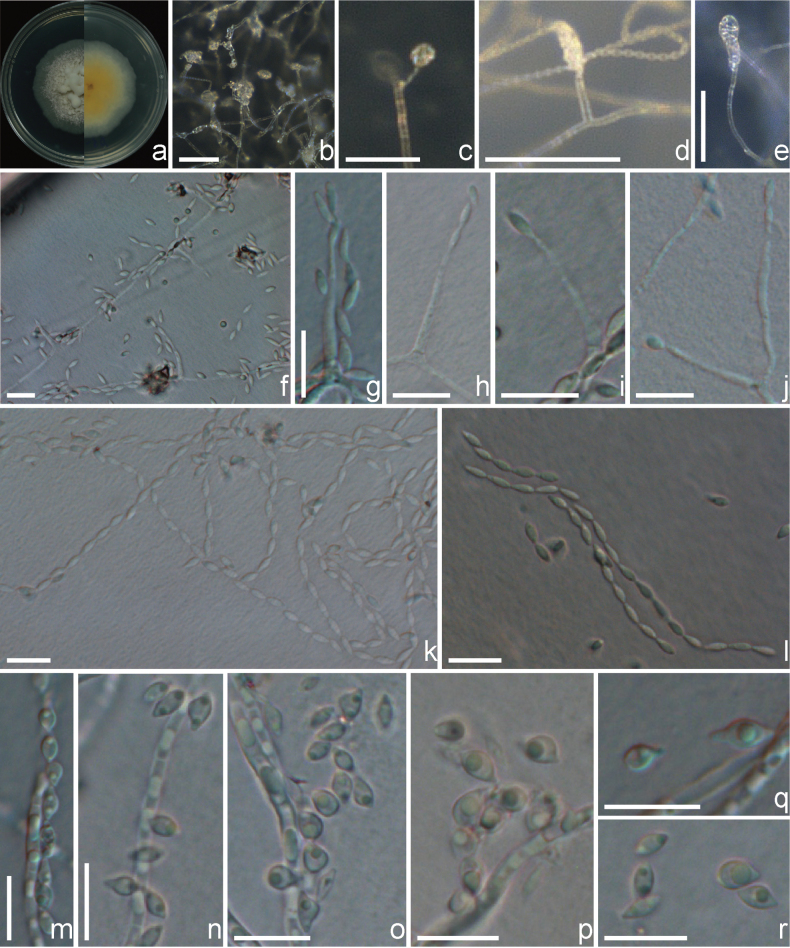
Morphology of *Sarocladium yunnanense* (WF8-2a1 = CFCC72677). a. PDA culture colonies; b–e. Conidiogenous structures and conidia on the colony; f. Conidiophores arranged on hyphae; g–j. Conidiophores; k, l. Conidial chains; m–r. Mature enlarged conidia. Scale bars: 50 μm (b); 50 μm (c); 20 μm (d); 10 μm (f–r).

##### Cultural characters.

Colonies on PDA 3.64–4.86 cm diam after 30 days at 25 °C, white to cinnamon, reverse yellowish white, flat, powdery; coarse flocculent in center, aerial mycelia occasionally seen near the edge; exudates not observed.

##### Additional strain examined.

China • Yunnan Province, Xishuangbanna Prefecture, Mengla County, Menglun Town, Xishuangbanna Tropical Botanical Garden (21°55'0.12"N, 101°15'E), dead leaves of the *Washingtonia filifera*, Nov. 2024, L.H. Zhu & D.W. Li, WF8-2a3 (CFCC72679).

##### Notes.

Phylogenetically, *Sarocladium yunnanense* formed an independent lineage in the *Sarocladium* clade, based on the ITS + LSU sequence data, and grouped with *S. subulatum*, *S. terricola*, and *S. bacillisporum* with strong support (99/1.00). In terms of the ITS sequence, the similarities (bp difference) between *S. yunnanense* and *S. subulatum*, *S. terricola* and *S. bacillisporum* are 93.43% (36 bp difference), 93.80% (34 bp difference), and 90.32% (49 bp difference), respectively. Whereas in the LSU, the similarities (bp difference) are 98.55% (8 bp difference), 987.32% (16 bp difference), and 97.49% (15 bp difference), respectively. Morphologically, these species can be easily distinguished from each other. The conidia of *S. yunnanense* become swollen and enlarged at a later stage, irregular or lemon-shaped with slightly truncate ends, while this does not occur in the other three species (Rambaut 2018). In addition, *S. yunnanense* can be clearly differentiated from *S. terricola* and *S. bacillisporum* by the colour of the colony, which is white in *S. terricola* and pinkish in *S. bacillisporum* (Gams and Hawksworth 1975).

## ﻿Discussion

*Cladosporium* s.s. and *Sarocladium* are well-studied fungal genera within Ascomycota, both morphologically and phylogenetically (Bensch et al. 2012; Giraldo et al. 2015; Ou et al. 2020; Hou et al. 2023; Lee et al. 2023). As cosmopolitan fungi with close associations with plant and human diseases, species of these genera have attracted considerable attention. At present, a robust taxonomic system for the two genera has been established, primarily based on the morphology of fruiting bodies formed naturally on hosts together with multilocus phylogenetic analyses (Rivera-Varas et al. 2007; Summerbell et al. 2011). On this basis, three new species isolated from palm leaves—*C. menglunense*, *S. menglaense*, and *S. yunnanense*—are described in this study based on both morphological characters and phylogenetic analyses.

The genus *Cladosporium* was initially recognized for its species being significant plant pathogens, such as *C. allii* (Ellis & G. Martin) P.M. Kirk & J.G. Crompton, which causes leaf blotch of onion (Jordan et al. 1990); *C. herbarum* (Pers.) Link, which contributes to cereal black point (Kryczyński and Weber 2011); and *C. cucumerinum* Ellis & Arthur, which is a major pathogen causing cucumber scab disease worldwide (Lee et al. 1997). Later research has shown that *Cladosporium* species are common across many regions and are ubiquitously detected in air, soil, and water; they are frequently isolated from indoor environments, public areas, and food products (Watson and Napier 2009). Based on integrated morphological and multilocus phylogenetic analyses, *Cladosporium* has been taxonomically defined into three major species complexes—*C. cladosporioides*, *C. herbarum*, and *C. sphaerospermum*—which are widely accepted by mycologists (Hamayun et al. 2009; Abdollahzadeh et al. 2020; Lee et al. 2023). Notably, most plant-related fungi of the genus are primarily associated with the *Cladosporium cladosporioides* complex. For instance, twelve *Cladosporium* species isolated from diverse plant hosts in China have been documented within this complex (Yang et al. 2023). However, the newly described *C. menglunense*, isolated from palm foliage in this study, phylogenetically clusters within the *C. sphaerospermum* complex. This discovery not only provides taxonomic novelty to the diversity of the complex but also adds novel mycological insights relevant to ecological adaptation.

Traditionally, species of *Sarocladium* have been reported as plant pathogens or as involved in human infections (Gams 1971; Chen et al. 1986; Helfer 1991). Many members of *Sarocladium* are associated with gramineous hosts, among which *S. attenuatum*, *S. oryzae*, *S. sinense* J.D. Chen, Guo C. Zhang & X.H. Fu, *S. sparsum* J.H. Ou, G.C. Lin & Chi Y. Chen, and *S. spirale* J.H. Ou, G.C. Lin & Chi Y. Chen were reported as causal agents of rice sheath rot (Chen et al. 1986; Ou et al. 2020). Some species, such as *S. bifurcatum*, *S. hominis* A. Giraldo, Gené & Deanna A. Sutton, *S. pseudostrictum* A. Giraldo, Gené & Deanna A. Sutton, and *S. subulatum* A. Giraldo, Gené & Guarro, have been isolated from human samples (Summerbell et al. 2011; Fernández-Silva et al. 2013; Sharma et al. 2013). This study represents the first report of *Sarocladium* species isolated from palm leaves, expanding the known host range of the genus beyond traditional gramineous plants and human clinical specimens. The discovery provides novel insights into the ecological adaptability of *Sarocladium* spp. across divergent plant hosts and underscores the importance of systematic surveillance of *Sarocladium* diversity in non-traditional hosts.

## Supplementary Material

XML Treatment for Cladosporium menglunense

XML Treatment for Sarocladium menglaense

XML Treatment for Sarocladium yunnanense

## ﻿Additional information

### Use of AI

No use of AI was reported.

### Author ORCIDs

Qiu-Yue Zhang https://orcid.org/0000-0001-9458-3566

Yu Wan https://orcid.org/0000-0002-2008-4745

De-Wei Li https://orcid.org/0000-0002-2788-7938

Hui Sun https://orcid.org/0000-0003-2015-6136

Li-Hua Zhu https://orcid.org/0000-0003-2740-4980

### Data availability

The sequences are deposited in the GenBank database (Tables 3, 4).
